# SARS-CoV-2 Spike Protein Extrapolation for COVID Diagnosis and Vaccine Development

**DOI:** 10.3389/fmolb.2021.607886

**Published:** 2021-07-28

**Authors:** Yashpal S. Malik, Prashant Kumar, Mohd Ikram Ansari, Maged G. Hemida, Mohamed E. El Zowalaty, Ahmed S. Abdel-Moneim, Balasubramanian Ganesh, Sina Salajegheh, Senthilkumar Natesan, Shubhankar Sircar, Muhammad Safdar, O. R. Vinodhkumar, Phelipe M. Duarte, Shailesh K. Patel, Jörn Klein, Parastoo Rahimi, Kuldeep Dhama

**Affiliations:** ^1^Division of Biological Standardization, ICAR-Indian Veterinary Research Institute, Bareilly, India; ^2^College of Animal Biotechnology, Guru Angad Dev Veterinary and Animal Science University, Ludhiana, India; ^3^Amity Institute of Virology and Immunology, Amity University, Noida, India; ^4^Department of Biosciences, Integral University, Lucknow, India; ^5^Department of Microbiology, College of Veterinary Medicine, King Faisal University, Hofuf, Saudi Arabia; ^6^Department of Virology, Faculty of Veterinary Medicine, Kafrelsheikh University, Kafr El-Shaikh, Egypt; ^7^Zoonosis Science Center, Department of Medical Biochemistry and Microbiology, Uppsala University, Uppsala, Sweden; ^8^Microbiology Department, College of Medicine, Taif University, Al-Taif, Saudi Arabia; ^9^Virology Department, Faculty of Veterinary Medicine, Beni-Suef University, Beni-Suef, Egypt; ^10^Laboratory Division, Indian Council of Medical Research - National Institute of Epidemiology, Ministry of Health & Family Welfare, Chennai, India; ^11^Young Researchers and Elites Club, Science and Research Branch, Islamic Azad University, Tehran, Iran; ^12^Faculty of Veterinary Medicine, Science and Research Branch, Islamic Azad University, Tehran, Iran; ^13^Indian Institute of Public Health Gandhinagar, Gandhinagar, India; ^14^Department of Breeding and Genetics, Cholistan University of Veterinary & Animal Sciences, Bahawalpur, Pakistan; ^15^Division of Epidemiology, ICAR-Indian Veterinary Research Institute, Bareilly, India; ^16^Veterinarian, Professor at the Faculty of Biological and Health Sciences, Universidade de Cuiabá, Primavera do Leste, Brazil; ^17^Division of Pathology, ICAR-Indian Veterinary Research Institute, Bareilly, India; ^18^Faculty of Health and Social Sciences, University of South-Eastern Norway, Kongsberg, Norway; ^19^Faculty of Veterinary Medicine, Science and Research Branch, Islamic Azad University, Tehran, Iran

**Keywords:** COVID-19, coronavirus pandemic, SARS-CoV-2, S-protein, diagnosis, vaccines

## Abstract

Severe acute respiratory syndrome coronavirus-2 (SARS-CoV-2) led to coronavirus disease 2019 (COVID-19) pandemic affecting nearly 71.2 million humans in more than 191 countries, with more than 1.6 million mortalities as of 12 December, 2020. The spike glycoprotein (S-protein), anchored onto the virus envelope, is the trimer of S-protein comprised of S1 and S2 domains which interacts with host cell receptors and facilitates virus-cell membrane fusion. The S1 domain comprises of a receptor binding domain (RBD) possessing an N-terminal domain and two subdomains (SD1 and SD2). Certain regions of S-protein of SARS-CoV-2 such as S2 domain and fragment of the RBD remain conserved despite the high selection pressure. These conserved regions of the S-protein are extrapolated as the potential target for developing molecular diagnostic techniques. Further, the S-protein acts as an antigenic target for different serological assay platforms for the diagnosis of COVID-19. Virus-specific IgM and IgG antibodies can be used to detect viral proteins in ELISA and lateral flow immunoassays. The S-protein of SARS-CoV-2 has very high sequence similarity to SARS-CoV-1, and the monoclonal antibodies (mAbs) against SARS-CoV-1 cross-react with S-protein of SARS-CoV-2 and neutralize its activity. Furthermore, *in vitro* studies have demonstrated that polyclonal antibodies targeted against the RBD of S-protein of SARS-CoV-1 can neutralize SARS-CoV-2 thus inhibiting its infectivity in permissive cell lines. Research on coronaviral S-proteins paves the way for the development of vaccines that may prevent SARS-CoV-2 infection and alleviate the current global coronavirus pandemic. However, specific neutralizing mAbs against SARS-CoV-2 are in clinical development. Therefore, neutralizing antibodies targeting SARS-CoV-2 S-protein are promising specific antiviral therapeutics for pre-and post-exposure prophylaxis and treatment of SARS-CoV-2 infection. We hereby review the approaches taken by researchers across the world to use spike gene and S-glycoprotein for the development of effective diagnostics, vaccines and therapeutics against SARA-CoV-2 infection the COVID-19 pandemic.

## Introduction

Severe acute respiratory syndrome coronavirus-1 (SARS-CoV-1), Middle East respiratory syndrome (MERS-CoV) and SARS-CoV-2 are among the highly pathogenic coronaviruses that infect humans (Jiang et al., 2020). Since the beginning of 21st century, animal coronaviruses (CoVs) have shown the ability to cross species barrier and infect humans to cause fatal illnesses. SARS-CoV-1 emerged in 2002 in Guangdong province of China and air travelers led to its spread to 29 countries in five continents (Drosten et al., 2003; Ksiazek et al., 2003) while MERS-CoV emerged in 2012 in Saudi Arabia and MERS is recorded in 27 different countries infecting about 2494 humans including 858 deaths (Zaki et al., 2012). In December 2019, a newly emerging SARS-CoV-2 (Huang et al., 2020; Zhu et al., 2020c was first reported in Wuhan, Hubei province of China (Zhou P. et al., 2020; Zhu et al., 2020c) and causes infections in humans in more than 210 countries in just a few months (Dhama et al., 2020a) leading to the first pandemic in history to be caused by a coronavirus.

SARS-CoV-2 infection causes atypical pneumonia and upper/lower respiratory tract infection that has affected nearly 71.2 million human cases and caused more than 1.6 million deaths in more than 215 nations as of 12 December 2020, and cases continue to rise as of the date of this publication with the highest number of cases in the United States where more than 16 M cases and 0.3 M deaths were reported. The World Health Organization (WHO) declared COVID-19 as a pandemic on March 11, 2020. The closely related viruses, SARS-CoV-1 and SARS-CoV-2, probably originated in bats which are a reservoir hosts of coronaviruses (Yang et al., 2016; Hu et al., 2017; Zhou P. et al., 2020). COVID-19 patients show common symptoms such as fever, cough, or chest tightness, and the majority have shown mild symptoms. However, some patients may develop severe disease with dyspnea and pneumonia while many patients report neurological manifestations as well as kidney damage (Guan et al., 2020; Dhama et al., 2020b). Using a mathematical model, the reproductive number (Ro) for SARS-CoV-2 is around 2.2. This suggests that one infected person can infect approximately 2.2 people. The higher Ro value for SAR-CoV-2 than other SARS-related viruses indicates more contagiousness.

**FIGURE 1:**
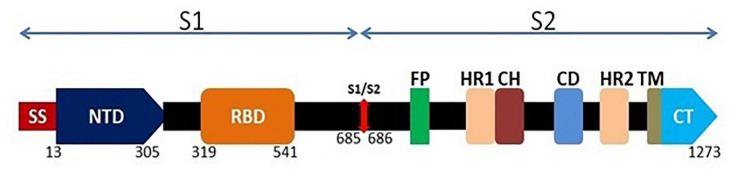
Schematic genomic organization of the full length spike glycoprotein of SARS-CoV-2. The surface spike protein contains S1 and S2 domain. At the N terminal, S1 domain consists of a signal sequence (SS) which is followed by N-terminal domain (NTD; 293 amino acids) and Receptor Binding Domain (RBD; 222 amino acids). S2 domain consists of fusion peptide (FP) followed by two heptad repeats (HR1 and HR2), a central helix (CH), a connector domain (CD), a transmembrane domain (TM) and the cytoplasmic tail (CT).

COVID-19 pandemic is continuous and rapidly spreading despite the aggressive measure in many parts of the world to contain SARS-CoV-2 transmission, leading to the urgent demand for development of rapid diagnostics including point-of-care testing while utilizing the advanced tools and techniques for effective and timely diagnosis, monitoring of the disease spread, contact tracing of the SARS-CoV-2 infections as well as addressing of limitations and challenges for designing different diagnostics (Afzal, 2020; Ai et al., 2020; Cheng et al., 2020; Dhama et al., 2020a; Dinnes et al., 2020; Kubina and Dziedzic, 2020; Natesan et al., 2020; Ravichandran et al., 2020; Tang et al., 2020; Udugama et al., 2020; Wu C. et al., 2020). Recently, a rapid test using automated platforms was approved, which involve high throughput automated tests providing results in 45–60 min (Uddin et al., 2020). Several molecular approaches are used for spike protein based diagnosis of SARS-CoV-2. SARS-CoV-2 infection in humans mostly occurs due to the interaction of the viral S-protein with angiotensin-converting enzyme 2 (ACE2) receptors on the host cell surface. The coronavirus S protein is a class I fusion The S-protein (Figure 1) consists of two subunits viz. S1 and S2; S1 has the receptor-binding domain (RBD) and S2 is involved in the fusion of virus membrane with the host cell membrane (Li, 2016; Walls et al., 2020). S-protein is cleaved by the host proteases between the S1 and S2 domains and the cleavage site is located upstream of the fusion domain. This cleavage activates membrane fusion protein through extensive irreversible conformational changes (Belouzard et al., 2009; Madu et al., 2009; Millet and Whittaker, 2015; Park et al., 2016). The host cellular proteases which cleave coronavirus spike proteins include proprotein convertases (e.g., furin), extracellular proteases (e.g., elastase), cell surface proteases [e.g., type II transmembrane serine protease (TMPRSS2)] and lysosomal proteases (e.g., cathepsin L and cathepsin B) (Millet and Whittaker, 2015).

A unique furin-like cleavage site (FCS) consisting of amino acid sequence PRRAR is found in the spike protein (S) of SARS CoV-2 and it appears to contribute to higher transmissibility and infectivity. This is absent in other B lineage of β CoVs such as SARS-CoV and other SARS-related bat coronaviruses such as the closest CoV RaTG13, which has 96% similarity with SARS-CoV-2 at the genomic sequence level. FCS is the potential cleavage site for furin proteases and this protease mediated cleavage of S-protein has significant implications on host susceptibility and zoonotic transmission of the virus. Mutations in this region may contribute to virus spread across the species barrier and results in spillover events. As the furin proteases are commonly present in the respiratory tract, the S glycoprotein is cleaved and then virus can enter into human respiratory tract epithelial cells and establish an infection (Andersen et al., 2020; Wrobel et al., 2020; Xia et al., 2020). These conserved regions of S-protein are extrapolated as potential targets for developing molecular diagnostic techniques. Further, the S-protein acts as an antigenic site for different serological assays employed for the diagnosis of COVID-19. SARS-CoV-2 infected humans may produce virus-specific IgM and IgG antibodies against the S-protein, which will bind to viral proteins in ELISA and lateral flow immunoassays.

The most effective method to prevent SARS-CoV-2 infection and an urgent need to the current COVID-19 pandemic is effective vaccine(s). There are continuous efforts in progress to develop an effective vaccine against SARS-CoV-2 infection. Different approaches are being tested and include inactivated and attenuated vaccines, viral vector-based vaccines, subunit protein and virus-like particle vaccines, DNA and RNA based vaccines (Al-Kassmy et al., 2020; Begum et al., 2020; Dhama et al., 2020c; Frederiksen et al., 2020; Rabaan et al., 2020; Yatoo et al., 2020). Each vaccine development platform and approach has its own advantages and disadvantages, and many of the vaccine platforms are simultaneously in different stages of clinical development (Amanat and Krammer, 2020). The S-protein of SARS-CoV-2 shows a very high amino acid sequence similarity to SARS-CoV-1, and the mAbs against SARS-CoV-1 cross-reacts with S-protein of SARS-CoV-2 and neutralizes its activity. Furthermore, *in vitro* studies have demonstrated that polyclonal antibodies targeted against the RBD of S-protein of SARS-CoV-1 can neutralize SARS-CoV-2 thus inhibiting its infectivity in permissive cell lines. This paves the way for the development of vaccines that may prevent newly emerging SARS-related CoVs and SARS-CoV-2 infections.

The exceedingly high mortality rates of severe and critical COVID-19 patients warrant the urgent need to identify and evaluate novel and specific antiviral therapeutics that could potentially prevent further clinical deterioration, reduce the need for advanced cardiorespiratory support and early mortality and mitigate the advanced disease manifestations. Few specific antiviral neutralizing mAbs targeted against SARS-CoV-2 such as Bamlanivimab [Drugs and Lactation Database (LactMed). Bethesda (MD): National Library of Medicine (United States); 2006–. Bamlanivimab. 2020 Nov 21. PMID: 33226744.] are in clinical development^1^. More recently, casirivimab [Drugs and Lactation Database (LactMed). Bethesda (MD): National Library of Medicine (United States); 2006–. Casirivimab. 2020 Nov 21. PMID:33226742.], and imdevimab [Drugs and Lactation Database (LactMed). Bethesda (MD): National Library of Medicine (United States); 2006–. Imdevimab. 2020 Nov 21. PMID:33226741] have received emergency use authorization on 21 November 2020 by the US FDA to treat mild to moderate COVID-19 in adults and pediatric patients^2^. Therefore, neutralizing antibodies (nAbs) targeting SARS-CoV-2 S-protein can be used for the pre-and post-exposure prophylaxis and in the immediate treatment of SARS-CoV-2 infection. This review highlights the recent updates on the use of S-protein-based diagnostics, vaccines and therapeutics to mitigate the ongoing devastating COVID-19 pandemic.

## S-Protein Based Diagnostics for SARS-CoV-2

### Molecular Diagnosis

**FIGURE 2:**
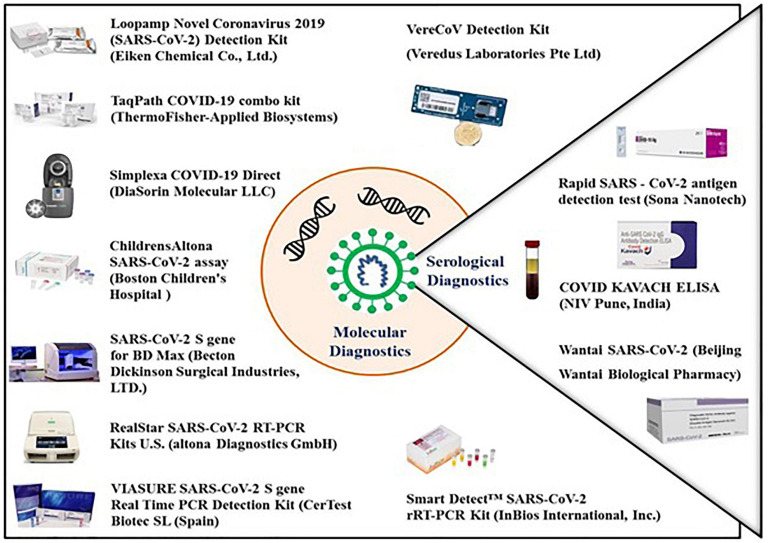
Molecular and serological diagnostic kits available for the detection of COVID-19. All the molecular diagnostic kits available as of the date of this publication are based on real-time RT PCR which detects the viral antigens with very high sensitivity as well as specificity. The serological kits are based on detection of antiviral specific antibodies and viral surface antigens. The antigen detection test kit identifies the viral surface protein in patient samples with the help of surface protein specific antibodies while the antibody detection kits identifies the viral surface protein specific IgG/IgM antibodies in the blood of an infected individual.

The detection of SARS-CoV-2 is currently based on viral nucleic acid detection using conventional and real-time RT-PCR assays using spike gene as a molecular target along with other genomic targets. Despite high selection pressure on SARS-CoV-2 spike protein, certain regions of the S protein remain widely conserved, including the S2 subunit and fragment of the receptor binding domain (RBD). These unique conserved regions in the spike gene can serve as a potential target in RT-PCR assays to give specific diagnostic results. Several molecular diagnostic tests targeting the spike gene have been developed as shown in Table 1 (Carter et al., 2020), which include the commonly used RealStar^®^ SARS-CoV-2 RT-PCR and the TaqPath COVID-19 combo assays as shown in Figure 2. The RealStar^®^ SARS-CoV-2 RT-PCR performs real time RT-PCR based qualitative detection of SARS-CoV-2 and can differentiate between betacoronavirus strains and SARS-CoV-2 specific viral RNA. The probes used in this real time PCR based assay is targeted to E gene of betacoronavirus and S-gene of SARS-CoV-2 which are labeled with FAM^TM^ fluorophore and Cy5 fluorophore, respectively, while JOE^TM^ fluorophore has been used to label the probe specific for an internal control (IC). Allplex^TM^ SARS-CoV-2 assay manufacture by Seegene is a multiplex assay that detects four different target genes that include gene encoding RdRP, S gene and N gene of SARS-CoV-2 and E gene of Sarbecovirus in Cal Red 610, Quasar 670, and FAM channel. This is highly compatible with the BioRad and other real-time PCR instruments.

**TABLE 1:** Molecular diagnostic assays used for the diagnosis of COVID-19.

Name of test	Assay type	Manufacturer	Type of sample	Target gene/region	Other information	Country of approval
Simplexa COVID-19 Direct assay	Real-time RT-PCR	DiaSorin Molecular LLC	Nasopharyngeal swabs	OFR1ab and S gene	Results in ∼1 h with no RNA extraction	United States
TaqPath COVID-19 combo assay	Multiplex real-time RT-PCR	Rutgers Clinical Genomics Laboratory Thermo Fisher Applied Biosystems	Oropharyngeal, nasopharyngeal, anterior nasal, midturbinate nasal swabs and saliva specimens	ORF1b and N and S genes		United States
RealStar SARS-CoV-2 RT-PCR assay	Real-time RT-PCR	Altona Diagnostics GmbH	Nasopharyngeal, oropharyngeal, anterior nasal, and mid-turbinate swabs, and nasal washes/aspirates	S gene		United States
SARS-CoV-2 S gene for BD Max	Real-time RT-PCR	Becton Dickinson Surgical Industries, LTD.	Nasopharyngeal and oropharyngeal swabs	S gene	Results for up to 24 samples in <3 h	Brazil
Childrens Altona SARS-CoV-2 assay	Real-time RT-PCR	Boston Children’s Hospital Infectious Diseases Diagnostic Laboratory (IDDL)	Nasopharyngeal, oropharyngeal, anterior nasal and mid-turbinate swabs, and sputum specimens	E and S genes		United States
VIASURE SARS-CoV-2 S gene Real-Time PCR assay	Real-time RT-PCR	CerTest Biotec SL (Spain)	Respiratory samples	S gene	Results in 2 h	Australia
Loopamp Novel Coronavirus 2019 (SARSCoV2) assay	Real-time RT-PCR	Eiken Chemical Co., Ltd.	Swabs and bronchoalveolar lavage fluid	ORF1ab and S gene	Results in ∼25 min	Japan
VereCoV Detection assay	Multiplex RT-PCR combined with microarray	Veredus Laboratories Pte Ltd.	Nasopharyngeal, nasal, throat swabs and throat aspirates	ORF1ab and N and S genes	Results in ∼2 h after extraction, lab-on-chip platform	Singapore
Allplex^TM^ SARS-CoV-2 assay	Multiplex real-time PCR	Seegene Inc.	Sputum, Nasopharyngeal swab, Nasopharyngeal aspirate, Bronchoalveolar lavage, Throat swab	E gene of Sarbecovirus, RdRP/S gene and N gene of SARS-CoV-2	Results in ∼1 h and 50 min after extraction	South Korea

Thermo Fisher Scientific has developed the *TaqPath COVID-19 Multiplex Diagnostic Solution* to enable clinical and public health laboratories to rapidly diagnose SARS-CoV-2 infection (Figure 2). The TaqPath COVID-19 Combo assay from Applied Biosystems (Thermo Fisher Scientific, United States) is a rapid diagnostic test based on real time RT-PCR which detects SARS-CoV-2 viral RNA with very high sensitivity. It screens 94 samples in less than 3 h or 382 samples in less than 6.5 h, with a single KingFisher purification system and a combination of Applied Biosystems real-time PCR system. The assay targets spike (S) gene, nucleocapsid (N) and Open reading frame-1ab (ORF-1ab) genes with a specificity of up to 100%.

Comparative analysis between representative molecular diagnostic tests approved for the detection of SARS-CoV-2 reveal that diagnostics based on spike gene as the target is comparable to other approved tools (Table 2).

**TABLE 2:** Comparison between RT-PCR based representative molecular diagnostic assays approved for diagnosis of COVID-19 (Kasteren et al., 2020).

Manufacturer	Target genes in SARS-CoV-2	LOD95
Altona Diagnostics	E	3.8
	S	3.8
Abbott Molecular Inc.	RdRp	3.1
	N	3.1
KH Medical	RdRp	4.8
	S	4.3
Primer Design	RdRp	23
SeeGene	E	4.8
	RdRp	18
R-Biopharm AG	E	4.3
BGI	RdRp	4.3

### Serological Diagnosis

Surface antigen on the spike protein of SARS-CoV-2 is an important target to develop serological assays for diagnosis of COVID-19. These assays detect spike protein specific antibodies in the serum of infected individuals. In an infected host, the immune system recognizes the exposed surface protein, especially protein S and E, and produces virus-specific IgM and IgG antibodies. Serum IgM and IgG will bind to viral proteins in ELISA and lateral flow immunoassay to confirm COVID-19.

As the genome of SARS-CoV-2 have nucleotide sequence similarity to the genome of SARS-CoV-1 and other common cold CoVs, there could be non-specific cross reactivity in the immunoassays. Thus, the specificity of spike protein based diagnostic immunoassays need to be validated. Using bioinformatics analysis, several novel antigenic epitopes of spike glycoprotein were identified and employed in developing SARS CoV-2 specific immunoassays (Deeks et al., 2020; Lokman et al., 2020). The recently published structure of the spike protein of SARS-CoV-2 can be used to identify the conformational arrangement of the epitopes in the spike protein for designing SARS-CoV-2 specific immunoassays and vaccines (Alam and Higgins, 2020; Walls et al., 2020).

The SARS-CoV-2 spike detection ELISA test has been developed for the quantitative detection of SARS-CoV-2 spike protein and is based on the solid phase sandwich enzyme immunoassay. This test contains antibodies specific for the recombinant spike protein and can recognize both the recombinant as well as wild-type spike protein with 67.02 pg/mL sensitivity. The LIAISON^®^ SARS-CoV-2 S1/S2 IgG test is another serological assay developed to detect the IgG antibodies specific for S1 and S2 antigens of SARS-CoV-2 (Figure 2). The test is highly efficient and can screen up to 170 samples in 1 h with a high sensitivity of up to 97% (beyond day 15) and a specificity of up to 98.5% to ensure accurate results. This test uses magnetic beads coated with S1 and S2 antigens expressed in human cells. Expression in human cells ensures proper folding and post-translational modifications of the recombinant antigens so that the antigens mimic the native spike protein on the surface of virus particle. Maintenance of the structure of S1 and S2 antigens ensures high specificity and enhanced concordance of this assay with the neutralization assay.

CDCs serologic testing includes the SARS-CoV-2 specific ELISA which uses perfusion stabilized form of SARS-CoV-2 spike protein to detect SARS-CoV-2 specific antibodies with a specificity of more than 99% and sensitivity of 96%. This assay can be used to diagnose prior SARS-CoV-2 infections without the need of confirmation with molecular diagnostic methods (Freeman et al., 2020). In this ELISA, minimum cross-reactivity is observed when antibodies against commonly circulating human coronaviruses are used.

Several diagnostic methods have been developed during the course of the COVID-19 pandemic. Among the developed methods, biosensor devices based on field effect transistor (FET) are highly advantageous due to their ability of rapid detection with high sensitivity which make them an ideal device to be used for point-of-care testing in clinical settings. Based on the characteristic features of graphene (large surface area, high conductivity and high carrier mobility), graphene based FET biosensors have been developed which are highly sensitive and specific in detecting environmental changes on its surface. In the study by Seo et al. (2020), a graphene-based FET biosensor device (COVID-19 FET sensor) was modified to detect SARS-CoV-2 using spike protein specific antibody which was immobilized on the device using probe linkers like 1-pyrenebutyric acid *N*-hydroxy succinimide ester (PBASE). The device could detect the viral antigen from different sources with a sensitivity of 1 fg/ml which supports its use in clinical settings. Furthermore, COVID-19 FET sensor was efficient enough to differentiate between SARS-CoV-2 and MERS-CoV antigens (Seo et al., 2020).

Several other serological assays have been developed to diagnose COVID-19 by detecting SARS-CoV-2 specific IgG and IgM in blood samples. Few serological tests use viral spike protein to diagnose viral infection as shown in the Table 2 (Carter et al., 2020). Of the available immunoassays for the detection of SARS CoV-2, the sensitivity of antibody detection tests is very low during the first few days of viral infection, however, it may have significance at later stage of the infection, when RT-PCR tests are negative (Deeks et al., 2020). Antigen detection can complement the RT-PCR detection assays during the first few days of SARS-CoV-2 infection; however, its sensitivity is much lower as compared to RT-PCR.

## S-Protein Based Vaccines for COVID-19

Infection by SARS-CoV-2 is initiated by binding of spike protein to ACE2 receptor on host cell surface, therefore, spike protein can be considered as a target for the development of a potential vaccine against COVID-19. Molecular analysis of the interaction between RBD of spike protein and the ACE2 receptor may further help in identification of antigenic sites and hence in the development of an effective vaccine as well as specific therapeutics for SARS-CoV-2. Genetic similarity between SARS-CoV-1 and SARS-CoV-2 could also be exploited for vaccine development and toward this end, numerous experimentally validated T cell and B cell epitopes, which were identical in SARS-CoV-1 and SARS-CoV-2 spike protein, have been identified by *in silico* approaches (Ahmed et al., 2020; Zheng and Song, 2020). Further the protective titer of neutralizing antibodies against SARS-CoV-2 is currently unknown and it needs to be revealed by animal challenge experiments with vaccine candidates.

In a study to develop a subunit vaccine against SARS-CoV-2, an immunogenic domain in the S2 subunit of spike protein which was conserved in SARS-CoV-1 and SARS-CoV-2 was identified and monoclonal antibodies specific for this conserved domain were produced which could identify the recombinant S-protein in addition to the S-protein in SARS-CoV-2 infected cells (Zheng et al., 2020).

The sequence similarity between the spike protein of SARS-CoV-1 and SARS-CoV-2 led to the generation of cross-neutralizing monoclonal antibodies using SARS-CoV-1 S-protein which may show the ability to neutralize SARS-CoV-2 (Zhou P. et al., 2020). CR3022 is one such neutralizing monoclonal antibody specific for SARS-CoV-1 RBD that could also bind to RBD of SARS-CoV-2 thus identifying an epitope that is not a part of receptor biding motif (RBM) (Tian et al., 2020). It has also been observed that the serum isolated from patients who have recovered from SARS could interfere with COVID-19 (Hoffmann et al., 2020). Polyclonal antibodies against SARS-CoV-1 RBD cross-neutralizes the RBD of SARS-CoV-2, therefore, neutralizing antibodies targeting SARS-CoV-1 RBD could be used for prophylaxis and treatment of COVID-19 (Tai et al., 2020). However, recent studies using plasma from SARS-CoV-1 or SARS-CoV-2 infected patients revealed that although antibodies show cross-reactivity, cross-neutralization is rare and non-neutralizing antibody response directed to conserved epitopes could be observed (Lv et al., 2020; Montelongo-Jauregui et al., 2020).

Studies have revealed that SARS-CoV-2 isolates from different parts of the world have similar amino acid sequences and hence, developed vaccines could be used worldwide to fight COVID-19. Several attempts were made previously to develop SARS-CoV-1 specific vaccine based on full length or partial spike protein and immunogenicity and protective efficacy of complete spike protein is well documented (Kam et al., 2007; Li et al., 2013). The spike protein in the form of recombinant protein or m-RNA have been tested as vaccine candidates at different doses in clinical trials. Tested the full-length spike protein at 5-μg and 25-μg doses, with or without Matrix-M1 adjuvant in 131 healthy adults by administering the vaccine in two intramuscular injections, 21 days apart. The results showed the spike protein-based vaccine is safe and it induced IgG titer above the levels observed in COVID-19 convalescent serum. Spike protein is also tested as in the form of mRNA as a vaccine candidate. Spike protein mRNA vaccine has been tested in a open-label trial including 45 healthy adults, administering two vaccinations of 28 days apart, at a dose of 25 μg, 100 μg, or 250 μg (15 participants in each dose group). The results showed that the mRNA vaccine elicited anti–SARS-CoV-2 immune responses in all participants without any trial-limiting safety concerns. The antibody response was found proportional to the dose of vaccine and systemic adverse events such as fatigue, chills, headache, myalgia, and pain at the injection site were more common among the individuals received highest dose and after the administration of second dose. However, vaccination using full length spike protein, antibody dependent enhancement (ADE) has also been reported in human monocytic or lymphoblastic cell lines (Kam et al., 2007; Jaume et al., 2012). This raises a concern over the use of full length spike protein as a vaccine target (Wang et al., 2020). RBD on the spike protein of SARS-CoV harbors several neutralizing epitopes which makes it a suitable vaccine candidate (He et al., 2005; Zhu et al., 2013). Experiments on mice model have revealed the ability of recombinant RBD based vaccine to induce long term protective immune response with significant level of SARS-CoV specific neutralizing antibodies (Du et al., 2007, 2009, 2010). Unlike full length spike protein, immunization with RBD based vaccine didn’t result in any pathogenic effect during SARS-CoV-1 infection (Kam et al., 2007; Jaume et al., 2012). Apart from RBD, other domains of spike protein viz. S1, S2, HR1 and HR2 domains have also been tested as vaccine candidates. Although, the neutralizing and protective immune response could be elicited using vaccines based on S1 fragment, HR1 domain and HR2 domain but induction of non-neutralizing antibodies could also be observed in mice vaccinated with S2 fragment (Guo et al., 2005; Li et al., 2013; Elshabrawy et al., 2012; Zheng et al., 2009; Wang et al., 2020). Based on the previous results and similarity between SARS-CoV-1 and SARS-CoV-2, it may be inferred that full length or partial spike protein could serve as good target for vaccine development against SARS-CoV-2.

Analysis of SARS-CoV-2 genomes isolated at the beginning of COVID-19 pandemic revealed the acquisition of D614G mutation in the spike protein. Viruses having this mutation had enhanced transmission efficiency and started to spread in Europe in early February 2020 but soon it became the dominant form of the virus worldwide (Korber et al., 2020; Zhang C. et al., 2020). In a study done by Weissman et al. (2020), it was shown that the D614G mutation in spike protein is not an escape mutation that would help the virus to evade the immune response generated against the vaccines that are currently being developed. Rather, G614 in the spike protein makes the virus more susceptible to neutralizing antibodies.

**FIGURE 3:**
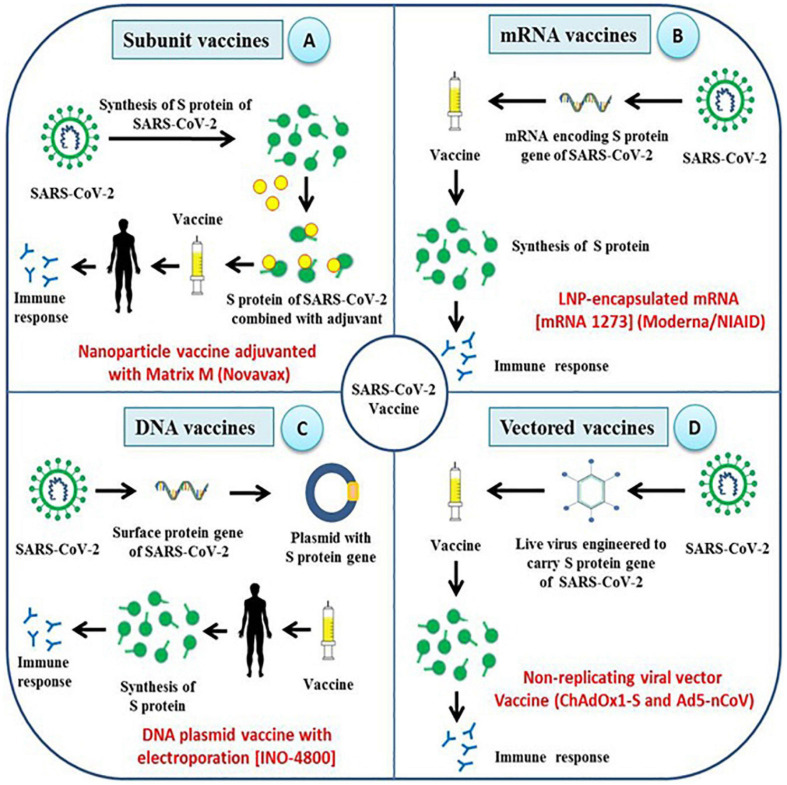
Different strategies employed for the development of vaccines against SARS-CoV-2. Research institutions along with pharmaceutical companies across the world are adopting unique strategies to develop effective vaccines against SARS-CoV-2 and most of them target spike proteins and its functional domains. **(A–D)** The type of vaccine being developed includes subunit vaccine, mRNA vaccine, DNA vaccine and viral-vector based vaccines which aim to elicit humoral as well as cellular immune response against spike protein of SARS-CoV-2.

Vaccine manufacturers and research institutions have taken initiatives to develop potential vaccine candidates to prevent SARS-CoV-2 infection in naive human population (Figure 3). The vaccine candidates which are currently under development aim to target the viral spike protein, which, being the surface protein, is important for causing infection and can also be the direct target of host immune response for neutralizing the virus (Wrapp et al., 2020). Potential regions of S protein that can be used as antigens include full length S protein, complete S1 subunit, RBD, NTD, CTD, and C-terminal membrane fusion domain of S2 subunit. Several vaccines against COVID-19 using spike protein as vaccine candidate are in different stages of clinical development, and their virus neutralization potential have been recently reviewed (Dong et al., 2020; Jeyanathan et al., 2020; Kaur and Gupta, 2020; Yuan et al., 2020). There are several neutralizing antibodies against the spike protein have also been developed for the use as therapeutic agents to treat COVID-19 patients (Jiang et al., 2020; Sharun et al., 2020; Zhou and Zhao, 2020).

## Selection of Viral Antigens for Vaccine Development

### Full-Length Spike Protein

The complete amino acid sequence maintains the correct conformation of a protein and hence provides maximum epitopes, thus exhibiting higher immunogenicity. Clover Biopharmaceuticals have patented their Trimer Tag Technology to construct a full-length SARS-CoV-2 S protein trimer vaccine (S-Trimer) (Liu et al., 2017; Zhang H. et al., 2020). They have proposed to produce the vaccine using a rapid mammalian cell culture-based expression system. Pre-clinical safety analysis is yet to be determined.

### Receptor Binding Domains (RBD)

Receptor binding domain of S protein directly interacts with the cell surface receptor, ACE2, and therefore, blocking the binding between RBD and ACE2 using specific antibodies could prevent virus infection. Moreover, RBD is highly conserved and contains multiple conformational neutralizing epitopes, which makes it a potential vaccine candidate (Jiang et al., 2005; Zhang H. et al., 2020).

### N Terminal Domain (NTD) of S1 Subunit

N terminal domain of S protein in several coronaviruses has been reported to have carbohydrate receptor binding activity (Krempl et al., 1997; Promkuntod et al., 2014). It has been observed that recombinant NTD of S protein from MERS-CoV could be used as a potent vaccine candidate to induce a cellular immune response in addition to antigen-specific neutralizing antibodies which was protective against wild type virus challenge (Chen et al., 2017; Jiaming et al., 2017). Although NTD of SARS-CoV-2 has not yet been evaluated, its use as a vaccine candidate may generate an effective immune response.

### Complete S1 Subunit

The S1 subunit contains NTD, CTD, and RBD which are responsible for initiating the infection process by SARS-CoV-2. A recombinant S1 subunit could provide a large number of neutralizing epitopes making the S1 subunit a good immunogenic candidate. Previously, MERS-CoV S1 subunit adjuvant vaccine has been evaluated to induce neutralizing antibodies having the potential to provide protection against wild type virus challenge (Wang et al., 2017; Adney et al., 2019).

### C-Terminal Membrane Fusion Domain of S2 Subunit

The fusion domain of the S2 subunit mediates the fusion of viral envelope with the host cellular membrane, and therefore, this domain could be a potential vaccine candidate. Currently, an RBD-fusion domain fusion protein was developed, which could induce high titer neutralizing antibodies in mice (Zhang H. et al., 2020).

## Spike Protein Based SARS-CoV-2 Vaccines Under Development

Various vaccines using different platforms including active immunization such as protein subunit vaccines, virus vectored vaccines, genetic vaccines (DNA or RNA), and monoclonal antibodies for passive immunization are under different stages of development (Yatoo et al., 2020).

### Subunit Vaccines

Subunit vaccines are safer and easy to produce, which often requires adjuvants for better immunogenicity. Several subunit vaccines are being developed (Figure 3). A SARS-CoV-2 specific subunit vaccine based on Molecular Clamp technology is being developed (Takashima et al., 2011), and Novavax, Inc. has developed nanoparticle vaccine candidate based on S protein which is under evaluation in animal models. Likewise, Johnson & Johnson, and Pasteur institute are also developing a subunit vaccine against SARS-CoV-2 (Zhang H. et al., 2020). Recently, Ravichandran et al. (2020) demonstrated the immunogenicity of the SARS-CoV-2 spike protein antigens which included S1 + S2 ectodomain, S1 domain, RBD and S2 domain. It was shown that all the antigens except S2 domain induced the production of neutralizing antibodies and RBD immunogen elicited the highest antibody titer compared to other tested antigens. Another study adopted a unique strategy to produce a recombinant fusion peptide wherein the RBD of SARS-CoV-2 S-protein was linked to mouse IgG1 Fc domain. The fusion peptide was shown to be significantly immunogenic and capable of stimulating protective humoral and cellular immune responses in vaccinated mice (Qi et al., 2020).

### mRNA Vaccines

mRNA vaccine represents an effective alternative to conventional vaccines due to its high potency, short production time, low cost, and safe administration (Pardi et al., 2018). Development of the mRNA vaccine includes antigen selection, optimization of sequences, screening of modified nucleotides, delivery optimization, evaluation of immune response, and safety check (Jahanafrooz et al., 2020). SARS-CoV-2 mRNA vaccine (mRNA-1273 encoding S protein) developed by Moderna has been evaluated in animal models and has also passed the Phase I clinical trial (Figure 3). An mRNA vaccine against SARS-CoV-2 is being developed and the mRNA used encodes either S-protein of SARS-CoV-2 or its RBD domain. In another approach, mRNA expressing SARS-CoV-2 virus-like-particles was used (Zhang H. et al., 2020).

### DNA Vaccines

DNA vaccines are DNA constructs encoding one or more viral antigens and are more suitable than mRNA vaccines in terms of stability and ease of administration (Liu, 2019). Some of the major disadvantages of using DNA vaccines include the chance of vector integration into host genome leading to possible mutations. Targeting SARS-CoV-2 using a DNA vaccine is one of the approaches adopted to combat COVID-19. One of the DNA vaccine candidate, INO-4800, which has been developed by INOVIO Pharmaceuticals, is expected to enter Phase I clinical trial (Smith et al., 2020) (Figure 3). Immunization of mice model with INO-4800 has been shown to induce highly significant T cell responses in addition to production of SARS-CoV-2 specific antibodies which could neutralize the virus and competitively inhibit the binding of virus to ACE2 receptors. Significant level of neutralizing virus specific IgG was also detected in BAL fluid of mice immunized with INO-4800 which shows that the vaccine could effectively be used as a prophylactic measure against SARS-CoV-2 infection. Recently, Yu et al. (2020) developed DNA vaccine candidates expressing six different variants of SARS-CoV-2 spike proteins which included full length spike protein, soluble ectodomain of S protein, S protein lacking the cytoplasmic and transmembrane domain, RBD of S protein, and S1 domain of S protein. Similarly, a DNA vaccine containing a mixture of different spike protein gene of different variant strains of SARS-CoV-2. As the virus spreads across the human and animal population, evolution of new variant strains is expected. Some variant strains with different mutations in spike proteins may produce different antibodies which may not confer cross protection against each other. A new variant strain of SARS-CoV-2 has been identified in farmed minks that is able to infect humans. This virus variant appears to be not effectively neutralized by the antibodies produced against the currently circulating SARS-CoV-2 (Oude Munnink et al., 2020). In such cases, the spike protein of different variant strains can be included in the DNA vaccine to provide broad spectrum protection against various SARS-CoV-2 strains. These factors need to be considered when developing a vaccine that can provide immunity against all the circulating virus strains, produce long lasting protective immunity, and can be used for universal vaccination. Challenge studies using the spike protein candidate vaccines in rhesus macaque model have shown the elicitation of both humoral as well as cellular immune responses in vaccinated individuals. DNA vaccine expressing the full length spike protein was most effective in reducing the viral load in BAL fluid and nasal mucosa (Yu et al., 2020). Several other candidate DNA vaccines have been developed and are in different stages of clinical development (Table 3).

**TABLE 3:** Serological assays using viral spike protein to diagnose SARS-CoV-2 infection.

Test/kit	Assay type	Manufacturer	Sample source	Target protein	Country of approval
Wantai SARS-CoV-2	Chromatographic lateral flow assay	Beijing Wantai Biological Pharmacy	Serum/plasma/whole blood	N protein, S1 and S2 subunits of S protein	Australia
Rapid SARS-CoV-2 antigen detection test	Antigen-based LFIA	Sona Nanotech	Nasal or oropharyngeal swabs	S1 domain of spike protein	Canada, Nova Scotia

### Vectored Vaccines

Viral vectors can be used to express heterologous antigens and are characterized by their high immunogenicity and safety. The SARS-CoV-2 specific vaccine based on the virus vector is under currently investigation and development (Zhang H. et al., 2020). A SARS-CoV-2 adenovirus vector vaccine has been constructed with the Greffex Vector Platform by Houston based Greffex Inc. and has been evaluated in animal models (Figure 3). Tonix Pharmaceuticals is also working in the development of the SARS-CoV-2 vaccine based on Horsepox virus (TNX-1800), and Johnson & Johnson has adopted the AdVac adenoviral vector platform for vaccine development (Gonzalez-Nicolini et al., 2006). In human trial using another adenoviral, Ad5 vectored COVID-19 vaccine was found tolerable and immunogenic following 28 days post-vaccination with stimulation of strong humoral responses and specific T-cell responses against SARS-CoV-2 (Zhu et al., 2020b). A randomized, double-blind, placebo-controlled, phase 2 trial of the Ad5-vectored COVID-19 vaccine administered at 5 × 10^10^ viral particles where around 508 eligible healthy adults aged 18 years or older was found safe, and induced significant immune responses in the majority of recipients following a single immunization (Zhu et al., 2020a). As any successful vaccine require a rigorous validation of safety and efficacy on the target population. In case of COVID-19 which is disproportionately affecting the elderly people with comorbidities, the vaccine should be effective in these population as well as the other populations including pregnant women, children and adults.

As of September 17, 2020, there are 27 candidate vaccines that are under clinical evaluation, and at least 64 candidate vaccines based on spike protein are under pre-clinical assessment around the world^3^. We, as a result of this, tabulate the candidate vaccines which are under clinical evaluation and use spike protein or its functional domain as immunogen (Table 4).

**TABLE 4:** COVID-19 candidate vaccines based on spike protein and its functional domains.

Vaccine platform	Vaccine candidate	Institution	Stage of clinical trial
Non-replicating viral vector	ChAdOx1-S	University of Oxford/AstraZeneca	Phase 3
Non-replicating viral vector	Adenovirus type 5 vector [Ad5-nCoV]	CanSino Biological Inc./Beijing Institute of Biotechnology	Phase 2
RNA	LNP-encapsulated mRNA [mRNA 1273]	Moderna/NIAID	Phase 3
Protein subunit	Full length recombinant SARS CoV-2 glycoprotein nanoparticle vaccine adjuvanted with matrix M	Novavax	Phase 1/2
RNA	3 LNP-mRNAs [BNT162]	BioNTech/Fosun Pharma/Pfizer	Phase 3
DNA	DNA plasmid vaccine with electroporation [INO-4800]	Inovio Pharmaceuticals	Phase ½
Protein Subunit	Recombinant RBD-dimer	Anhui Zhifei Longcom Biopharmaceutical/Institute of Microbiology, Chinese Academy of Sciences	Phase 2
Non-replicating viral vector	Ad26COVS1	Janssen Pharmaceutical Companies	Phase ½
Protein subunit	RBD based	Kentucky Bioprocessing, Inc.	Phase ½
Protein subunit	S protein (baculovirus production)	Sanofi Pasteur/GSK	Phase ½
Protein subunit	Native like trimeric subunit spike protein vaccine (SCB-2019)	Clover Biopharmaceuticals Inc./GSK/Dynavax	Phase 1
Protein subunit	Recombinant spike protein with Advax^TM^ adjuvant	Vaxine Pty Ltd./Medytox	Phase 1
Protein subunit	Molecular clamp stabilized spike protein with MF59 adjuvant	University of Queensland/CSL/Seqirus	Phase 1
Protein subunit	S-2P protein + CpG 1018	Medigen Vaccine Biologics Corporation/NIAID/Dynavax	Phase 1
Non-replicating viral vector	Replication defective simian adenovirus (GRAd) encoding S	ReiThera/LEUKOCARE/Univercells	Phase 1
Protein subunit	RBD plus adjuvant	Instituto Finlay de Vacunas, Cuba	Phase 1
Protein subunit	RBD (baculovirus production expressed in Sf9 cells)	West China Hospital, Sichuan University	Phase 1
Non-replicating viral vector	Adeno-based (rAd26-S + rAd5-S)	Gamaleya Research Institute	Phase 1
Replicating viral vector	Intranasal flu-based-RBD	Beijing Wantai Biological Pharmacy/Xiamen University	Phase 1
DNA	DNA vaccine (GX-19)	Genexine Consortium	Phase 1
RNA	mRNA	Curevac	Phase 1
DNA	DNA plasmid vaccine + adjuvant	Osaka University/AnGes/Takara Bio	Phase 1
RNA	mRNA	Arcturus/Duke-NUS	Phase ½
Replicating viral vector	Measles-vector based	Institute Pasteur/Themis/University of Pittsburg CVR/Merck Sharp & Dohme	Phase 1
RNA	LNP-nCoVsaRNA	Imperial College London	Phase 1
RNA	mRNA	People’s Liberation Army (PLA) Academy of Military Sciences/Walvax Biotech	Phase 1
DNA	DNA plasmid vaccine (ZyCov-D)	Cadila Healthcare Limited	Phase ½

We also tabulate the advantages and disadvantages of various approaches for vaccine development (Table 5).

**TABLE 5:** Comparison between different approaches to develop SARS-CoV-2 vaccines using S protein or whole virus.

Types of vaccines	Advantages	Disadvantages
Subunit vaccines	✓ Protection against viral infection	✓ May have limited efficacy ✓ Lead to imbalanced immune response
mRNA vaccines	✓ Rapid development ✓ Low cost manufacture	✓ Cellular delivery and distribution in organs affected by properties of RNA ✓ Unknown safety among human beings
DNA vaccines	✓ Enhances humoral and cellular immune response ✓ Stable ✓ Easy to prepare and harvest in large quantity	✓ Unknown safety and efficacy for use in human beings
Vectored vaccines	✓ Can infect APCs directly ✓ Physically and genetically stable	✓ May induce poor immunity to vector
Whole virus inactivated vaccine	✓ Easy to produce ✓ Stable expression of conformational epitopes ✓	✓ Unimportant antigen may skew the immune response ✓ Need BSL3 facility for growth of pathogen

## Development of Therapeutics Targeting Spike Protein and Its Function

Therapeutic strategies targeting the spike protein of SARS-CoV-2 are valuable for developing specific antiviral drugs. Computational screening of drug libraries for potential targets on spike protein of SARS-CoV-2 followed by repurposing of the identified drugs will help in rapid discovery of COVID-19 specific antiviral small molecule inhibitors (Zhou Y. et al., 2020). A study on virtual screening of small molecules against the viral spike protein revealed that several synthetic and natural compounds have a high binding affinity with the spike protein. However, hesperidin was the only compound capable of targeting the binding interface between spike and ACE2 (Wu S. Y. et al., 2020). In another study, researchers demonstrated the use of TMPRSS2 serine protease by SARS-CoV-2 for entry into host cell and S protein priming (Hoffmann et al., 2020). Now there is an approved TMPRSS2 inhibitor for clinical use that blocked the entry of SARS-CoV-2 and might constitute a treatment option for future and can be identified a potential target for antiviral intervention (Hoffmann et al., 2020).

In addition, the N-terminus of SARS-CoV-2 has a ganglioside binding domain which can be mimicked by drugs such as chloroquine and hydroxychloroquine (Fantini et al., 2020). Mouse studies have also shown that enhanced expression of soluble ACE2 protein in the presence of angiotensin receptor blockers can inhibit SARS-CoV-2 infection in susceptible host cells and mitigate damage to host tissues. Lei et al. (2020) demonstrated the inhibition of propagation of SARS-CoV-2 in engineered human tissues by the use of soluble recombinant ACE2.

Small molecule inhibitors can also be a significant in preventing the spread of SARS-CoV-2 infection. In several previous studies, these inhibitors were found to block the interaction between SARS-CoV and ACE2 receptor on the host cell surface thus preventing virus infection (Huentelman et al., 2004; Kao et al., 2004; Adedeji et al., 2013). Similar strategies could be used to inhibit SARS-CoV-2 infection in susceptible host cells using small molecule inhibitors. In a drug repurposing screening using a proximity-based AlphaLISA assay, which measures binding of S-protein RBD with ACE2, 25 out of 3384 small molecule drugs could be identified to be evaluated for its efficacy against SARS-CoV-2 (Hanson et al., 2020).

## Conclusion and Prospects

COVID-19 is a health crisis facing humans in the 21st century. The current global efforts aim to contain the COVID-19 pandemic, minimize the rate and number of infections, reduce the burden on hospitals, and to minimize the socio economic impact. The current data on new COVID-19 cases and their characteristics will provide information on the modeling of new cases in future and planning for healthcare capacity. Until SARS-CoV-2 vaccine(s) are available for clinical use in humans, the adopted mitigation strategies will help reduce the impact of the ongoing coronavirus pandemic and its negative consequences on human health and activities. Currently, many vaccines are in different developmental stages using several vaccine platforms. SARS-CoV-2 have many pathologic similarities with previous human highly pathogenic SARS-CoV and MERS-CoV, which may help understand the immune response and develop a safe and effective vaccine. Currently, there no vaccines against CoVs in clinical use in humans and animals. Most of these vaccine development strategies target the S-protein of SARS-CoV-2. Likewise, antiviral therapeutic strategies target the S-protein of SARS-CoV-2 may lead to the development of specific antiviral agents to prevent SARS-CoV-2 infections. The vaccine for SARS-CoV-2 may not be available for the first wave of pandemic but will be helpful if the second or third waves exits or if SARS-CoV-2 become a seasonal human CoV. Future coordinated plans are required to be adopted to develop preclinical vaccine candidates more efficiently, which may be achieved by coordination between governmental sectors, health authorities, pharmaceutical manufacturers, regulatory agencies, and the WHO. Further, the lessons learned from the ongoing COVID-19 pandemic will help prepare for newly emerging zoonotic viruses of pandemic potential in the future.

## Author Contributions

YM, PK, MA, and MEZ contributed to the conceptualization of the manuscript and initial data collection. BG, SiS, SN, MH, MEZ, and ShS contributed to the initial draft writing, editing and updating the information. MS, OV, PD, SP, and AA-M contributed to the design and generation of the figures. JK, PR, MEZ, and KD contributed to editing of the manuscript. YM supervised the work, compiled all data, and edited and submitted the manuscript. All authors listed have made a substantial, direct and intellectual contribution to the work, and approved it for publication. All authors contributed to the article and approved the submitted version.

## Conflict of Interest

The authors declare that the research was conducted in the absence of any commercial or financial relationships that could be construed as a potential conflict of interest.

## Publisher’s Note

All claims expressed in this article are solely those of the authors and do not necessarily represent those of their affiliated organizations, or those of the publisher, the editors and the reviewers. Any product that may be evaluated in this article, or claim that may be made by its manufacturer, is not guaranteed or endorsed by the publisher.

## Footnotes

1
https://clinicaltrials.gov/ct2/show/study/NCT04411628

2
https://www.fda.gov/news-events/press-announcements/coronavirus-covid-19-update-fda-authorizes-monoclonal-antibodies-treatment-covid-19#:~:text=Monoclonal%20antibodies%2C%20such%20as%20casirivimab,health%20during%20this%20unprecedented%20pandemic

3
https://www.who.int/publications/m/item/draft-landscape-of-covid-19-candidate-vaccines
